# Method to Determine the Optimal Aptamer-to-Bead Ratio by Using Flow Cytometry

**DOI:** 10.1155/2023/5842652

**Published:** 2023-07-11

**Authors:** Sun Young Lee, Eun-Ok Kim, Daehyuk Jang, Soonjae Hwang, Ki-Jong Rhee, Miyong Yun

**Affiliations:** ^1^Lab of Functional Aptamer, Department of Bioindustry and Bioresource Engineering, College of Life Sciences, Sejong University, Seoul, Republic of Korea; ^2^Resource Upcycling and Discovery Research Institute, Sejong University, Seoul, Republic of Korea; ^3^Medical Science Research Center, Korea University College of Medicine, Seoul, Republic of Korea; ^4^Department of Biochemistry, Lee Gil Ya Cancer and Diabetes Institute, College of Medicine, Gachon University, Incheon, Republic of Korea; ^5^Department of Biomedical Laboratory Science, College of Software & Digital Healthcare Convergence, Yonsei University MIRAE Campus, Wonju, Gangwon-do, Republic of Korea

## Abstract

Research on the effective attachment of aptamers to beads, which is essential for using aptamers, has made relatively little progress. Here, we demonstrate a new method based on flow cytometry to determine the optimal aptamer-to-bead ratio for aptamer immobilization. The fluorescence intensity increased with a gradual two-fold increase in the aptamer fluorescence concentration, peaked at an aptamer-to-bead ratio of 2.56 × 10^5^, and tended to decrease at higher ratios. A similar pattern was observed in an additional analysis using fluorescence microscopy. However, measurement of the free aptamer concentration after the aptamer-bead conjugation reaction revealed a large aptamer loss compared to the 1.28 × 10^5^ aptamer-bead ratio. In addition, the binding efficiency of the aptamer/bead to the target was highest at the aptamer-to-bead ratio of 1.28 × 10^5^. Taken together, our data suggest that the proposed method is the best and easiest for determining the optimal aptamer-to-bead ratio.

## 1. Introduction

Flow cytometry is a popular cell analysis technique used in various fields. It was first used in the 1950s to measure the volume of cells in a rapidly flowing fluid stream and was then developed to analyze cell characteristics [1]. The technique is predominantly used to measure the fluorescence intensity produced by fluorescently labeled antibodies to detect target proteins. In addition, the technique has been used to recognize nonfluorescent cells by side and forward scatter through the detection of scattering at a 90° angle relative to the laser and along the path of the laser, respectively [2]. The greatest advantage of flow cytometry is the high reliability of the results, which allows the rapid analysis of multiple samples and easy comparison with the control group [3]. In recent years, flow cytometry has been applied in various ways other than for cell analysis. For example, flow cytometry has been used to compare and measure the amounts of various proteins in cells [4]. It has also been used to quantify secreted proteins such as cytokines using beads with many different fluorescently labeled antibodies at the same time [5, 6].

Three independent groups of aptamers have been identified. The term “aptamer” refers to a short single-strand nucleotide (DNA or RNA) or peptide that can specifically bind to the desired target [7–9]. Owing to the high specificity of aptamers to their target molecules, aptamers have found applications in various fields such as biosensing, diagnostics, and therapeutic applications [10, 11]. Various techniques for the systematic evolution of ligands by exponential enrichment (SELEX) have been used in many studies to discover aptamers that interact with specific targets [12]. In the following study, aptamers discovered through SELEX should be attached to appropriate beads, either by passive adsorption or covalent coupling depending on the intended application [13]. The beads are available in a variety of formats (polystyrene, agarose, and magnetic beads) and are coated with carboxyl groups, avidin, streptavidin, or other proteins. Magnetic beads coated with a linker arm for immobilization were first used in 1997 [14]. The coated beads can be used in various applications. For example, carboxyl-coated polystyrene beads have been used for binding to amine-modified aptamers and streptavidin-coated agarose beads have been used for binding to biotin-labeled antibodies [15]. Currently, aptamers are attached to beads either chemically or through a specific interaction such as that between avidin and biotin.

However, comprehensive studies to determine the optimal aptamer-to-bead ratio have not yet been reported. This motivated our study, in which we used flow cytometry to determine the optimal amount of aptamer to form the aptamer-bead complex.

The aforementioned complex is essential for aptamer studies. Some papers provide information about the reagents, but the molar ratio bead-aptamer is ill-defined [16]. Furthermore, most papers do not indicate the exact amount or percentage of aptamers and beads used [17]. The optimal binding ratio of the aptamer is different for each experiment and depends on the binding method and size of the beads. Therefore, it is important to determine the optimal aptamer-to-bead binding ratio in different experimental environments.

## 2. Materials and Methods

### 2.1. Aptamer and Other Chemicals

RNA aptamer N1 (NH_2_-5′-GGGAGAGGAUACUACACGUGAUAGUCAGGGAACAUG ACAAACACAGGGACUUGCGAAAAUCAGUGUUUUGCCAUUGCAUGUAGCAGAAGCUUCCG-3′) (Supplementary Table) with or without FAM (5′-Fluorescein phosphoramidite), selected as a specific aptamer binding Ni^2+^/Co^2+^ [18], were synthesized by Integrated DNA Technologies (IDT). This aptamer was selected because the average length of the metal ion-specific aptamer is 80–100 m, and the available equipment could be used to confirm the amount of Ni^2+^/Co^2+^ bound to the aptamer. The amine group at the 5′-terminus was designed to allow covalent binding of the aptamer to the carboxyl groups on the CLB9 beads (Sigma), which were used to immobilize the aptamers. MES buffer (50 mM, pH 5.9) was used as the solvent in which to couple the aptamer to the beads and to subsequently wash the beads. For coupling, the carboxyl groups on the surface of the beads were activated using N-(3-dimethylaminopropyl)-N-ethylcarbodiimide (EDC) (Sigma) and N-hydroxysulfosuccinimide (sulfo-NHS) (G-Biosciences). Cobalt (II) chloride was purchased from Sigma–Aldrich (St. Louis, MO, USA; 449776-5G).

### 2.2. Aptamer-Bead Complex

To prepare the aptamer-bead complex, a 50-*μ*L (2.5 × 10^11^, 0.9 *μ*m mean bead size) aliquot of beads was transferred to a microtube and centrifuged for 4 min at 13,000×*g*. The supernatant was carefully removed and discarded. The bead pellet was resuspended in 200 *μ*L MES buffer and vortexed thoroughly for 10 s. To activate the bead surface, 40 *μ*L of 0.3 M EDC and 40 *μ*L of sulfo-NHS 0.4 M solution were added, followed by mixing. After activation, different concentrations of the aptamer were added to the microtubes and the total volume was adjusted to 500 *μ*L. The coupling reaction was performed for 2 h in the dark at room temperature with rotation (Roto-Bot; Benchmark, USA). The tubes were centrifuged for 4 min at 13,000×*g*. The supernatant was used to measure the amount of free aptamer, and the pellet was resuspended in 500 *μ*L PBS-T. This washing step was repeated thrice.

### 2.3. Free Aptamer Measurement

The fluorescence of the supernatants was measured using a Tristar 5 plate reader (Berthold, Germany). Absorbance data were collected and processed using the MikroWin2010 program (Labsis Laborsysteme GmbH). The manufacturer's instructions were followed for operating the machine.

### 2.4. Flow Cytometry Analysis

Beads with or without aptamer labeled with FAM were suspended in a total volume of 300 *μ*L PBS, followed by analysis using the FACS Canto II flow cytometer (BD Biosciences, CA, USA). Data were collected using FACSDiva software (BD Bioscience, CA, USA) and subsequently analyzed using FlowJo software (FlowJo, OR, USA).

### 2.5. Fluorescence Image Capture

The fluorescently labeled aptamer-bead complex was analyzed using a CKX53 microscope (Olympus, Tokyo, Japan). After aptamer-bead coupling, a 5 *μ*L aliquot of beads was transferred onto a slide glass and covered with the cover glass. The microscope was controlled by a computer with the CellSens Entry software (Olympus) at 200× magnification. Photographic images were captured in a completely darkened room, and the entire process was completed within 10 minutes.

The fluorescence intensity on the images was measured using ImageJ software (National Institutes of Health, MD, USA). First, Photoshop (Adobe Systems, CA, USA) was used to select regions of interest (ROIs), measuring 100 × 100 pixels from the images. ROIs of the same size were selected and cut with Photoshop in the same positions in all images to maintain a standard in the analysis of the measurements. Next, ImageJ software was used to extract the average brightness value from the ROI. Each pixel was assigned a numerical value that represented its brightness on the grayscale 22, ranging from 0 (black pixels) to 255 (white pixels).

### 2.6. ICP-OES Measurements

After aptamer–bead coupling, 300 *μ*L of 10 *μ*M CoCl_2_ was transferred to a microtube containing the aptamer–bead complex and the mixture was incubated for 10 min at room temperature with rotation (Roto-Bot) [19]. After incubation, the microtube was centrifuged for 4 min at 13,000×*g*, and the concentration of Co^2+^ in the supernatant was measured by ICP-OES using a PlasmaQuant PQ 9000 spectrometer (Analytik Jena, Germany) equipped with a Standard Kit. The data were collected and processed using Aspect PQ 1.2.4.0 software. The manufacturer's instructions were followed for operating the machine.

### 2.7. Statistical Analysis

All statistical analyses were conducted using SPSS software (IBM® SPSS® Statistics Ver. 23). For multiple comparisons, the data were analyzed using one-way analysis of variance (ANOVA). Tukey's posthoc test was used to identify significant differences among groups.

## 3. Results and Discussion

### 3.1. Flow Cytometry Can Detect Beads to Which Aptamers Are Attached but Not Those without Aptamers

**Figure 1:**
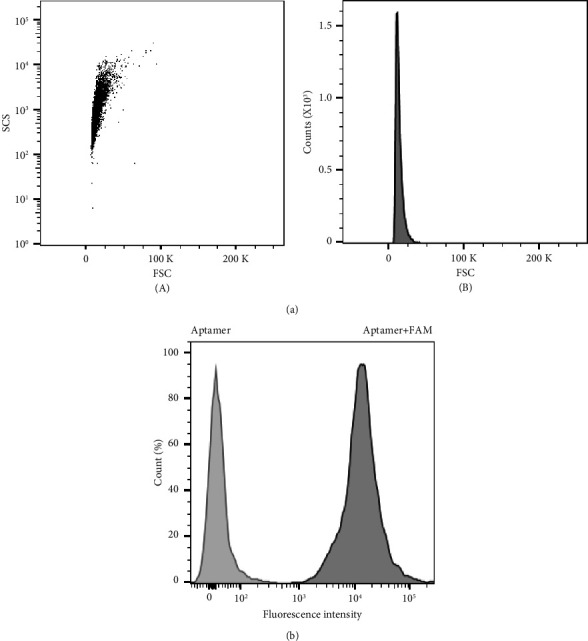
Flow cytometry analysis of aptamer-bead complexes. Results of the analysis of 1 × 10^4^ beads after interaction with the aptamer or fluorescent aptamer. (a) Scatter plot (A) and histogram (B) of the aptamer-bead complex without fluorescent labeling. FSC: forward scatter, SSC: side scatter, *K*: × 10^3^. (b) Comparative histogram of beads that had interacted with the aptamer or fluorescent aptamer. FAM: 6-carboxyfluorescein.

Flow cytometry, in which the laser passes through the cells and is reflected, uses pattern analysis to acquire information about the characteristics of cells. To explore whether flow cytometry could recognize polystyrene beads, we started our investigation by analyzing the beads without and with aptamers attached. Flow cytometry did not recognize the beads without aptamers; however, those with aptamers were detected (Figure 1(a)). Specifically, polystyrene beads to which fluorescently labeled aptamers were attached, were clearly detected (Figure 1(b)). In general, the way flow cytometry perceives cells is to analyze three parameters depending on the degree of light scattering when the cell passes through the laser beam [3, 20]. In other words, the front-facing light collecting forward scatter (FSC) represents the size of the cell, and the side scatter (SSC) collecting light from the side represents the intracellular granularity, and finally, the fluorescence intensity [21]. Beads without an aptamer are analyzed by FSC, which entails detecting the laser light scattered from the front; however, because SSC, which is the lateral light used to measure the presence of granules in the cell, is not generated, these beads are not necessarily recognized by flow cytometry. However, in the case of an aptamer-coupled bead, flow cytometry would be able to recognize it because the light is diffusely reflected by the aptamer and collected by detecting the SSC.

### 3.2. Determination of the Aptamer-to-Bead Ratio by Flow Cytometry

**Figure 2:**
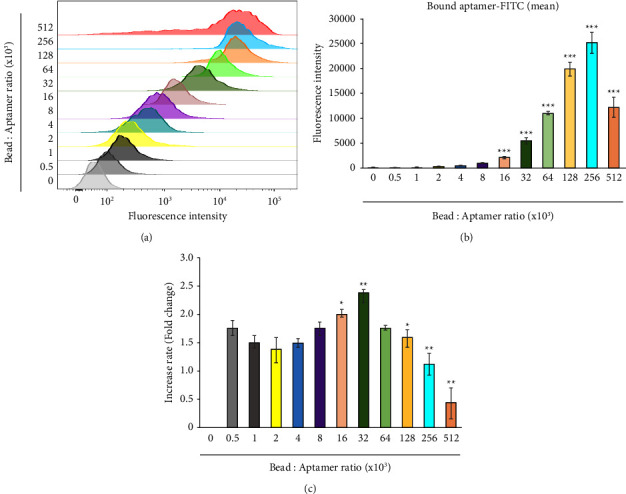
Determination of the ratio of aptamer and bead concentrations by flow cytometry. Beads were coupled with aptamer_FAM at ratios gradually increasing from 0.5 × 10^3^ to 5.12 × 10^5^. (a) Histogram overlay of fluorescence intensity of gradually increased aptamer-FAM. FAM: 6-carboxyfluorescein. (b) Bar graphs representing the fluorescence in (a). “0” indicates control without aptamer. All experiments were repeated three times. Error bars represent standard deviations of replicates (*n* = 3). ^*∗∗∗*^Statistically significant difference in fluorescence intensity compared to control (*P* value <0.001). (c) Bar graph representing the rate of fluorescence increase obtained by dividing the average of the indicated ratio by the previous average fluorescence. Statistically significant difference in fluorescence intensity compared to ratio 0.5 × 10^3^. ^*∗*^*P* < 0.05, ^*∗∗*^*P* < 0.01. Without a star, no statistical significance.

Because various types of beads are used in research with aptamers, it is inefficient to use the same aptamer-to-bead ratio for all beads. Therefore, determining the most effective ratio for each type of bead is essential. In our work, the aptamer-to-bead ratio was gradually increased from 5 × 102 to 5.12 × 105 by doubling the aptamer concentration incrementally while the concentration of beads was kept constant. As expected, the fluorescence intensity increased approximately 2-folds with a 2-fold increase in the aptamer concentration (Figures 2(a) and 2(b)). This analysis indicated that the aptamer-to-bead ratio of 2.56 × 105 was the most effective. However, interestingly, the fluorescence intensity tended to decrease at the higher ratio of 5.12 × 105 (Figures 2(a) and 2(b)). The rate at which the fluorescence intensity increased became slower at the 6.4 × 104 ratio (Figure 2(c)). This result means that, above a specific concentration of the aptamer (in this case, 6.4 × 104), the efficiency whereby the aptamer binds to the bead is diminished. This may be due to the possible formation of nonspecific conjugates between the larger number of aptamer molecules in a fixed volume of buffer. Taken together, these results indicate that the optimal aptamer-to-bead ratio is between 6.4 × 104 and 2.56 × 105.

### 3.3. Confirmation of the Aptamer-to-Bead Ratio by Fluorescence Microscopy

**Figure 3:**
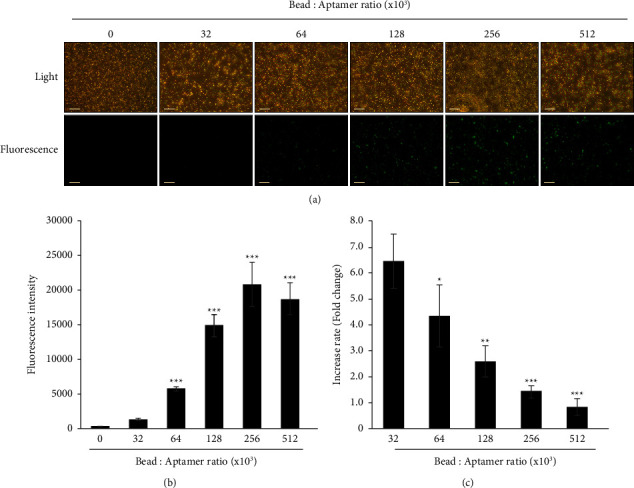
Confirmation of aptamer-to-bead ratio by fluorescence. (a) Fluorescence microscopy analysis of beads coupled with aptamer_FAM at ratios gradually increasing from 0 to 256 × 10^3^ (scale bar = 20 *μ*m). Con: light microscopy, FAM: fluorescence microscopy (green). (b) Bar graphs representing the fluorescence in (a). Photomicrographs were taken at 200× magnification. “0” indicates control without aptamer. All experiments were repeated three times. Error bars represent standard deviations of replicates (*n* = 3). ^*∗∗∗*^Statistically significant difference in fluorescence intensity compared to control (*P* value <0.001). (c) Bar graph representing the rate of fluorescence increase obtained by dividing the average of the indicated ratio by the previous average fluorescence. The statistically significant difference in fluorescence intensity compared to ratio 32 × 10^3^. ^*∗*^*P* < 0.05,^*∗∗*^*P* < 0.01,^*∗∗∗*^*P* < 0.001. Without a star, no statistical significance.

To confirm the aptamer-to-bead ratio we determined by flow cytometry, we used fluorescence microscopy to analyze complexes containing fluorescently labeled aptamers and beads. The fluorescence intensity gradually increased under the same conditions as those in Figure 2 (Figures 3(a) and 3(b)). An aptamer-to-bead ratio of 2.56 × 105 was found to be optimal, similar to the ratio determined using flow cytometry. We also confirmed that the fluorescence intensity decreased at higher ratios of 5.12 × 105, similar to the results obtained by flow cytometry (Figure 3(b)), and that the decrease was not excessive, similar to that observed by flow cytometry. This difference may be due to the fact that the samples measured using flow cytometry are fast flowing, which would result in all nonspecifically bound aptamers becoming dislodged from the beads. Unlike flow cytometry analysis, however, the rate at which the fluorescence intensity increased started declining at an earlier point, i.e., at a ratio of 3.2 × 104 (Figure 3(c)). In the case of fluorescent images acquired using confocal microscopy, it is difficult to compare the exact intensity of light in different samples. This is because the overall brightness of the image is adjusted according to the intensity of the bright light. Therefore, the correction of the light intensity would have been applied from the ratio of 3.2 × 104 of which the image contains strong fluorescent spots (Figure 3(a)). Based on these data, the most effective aptamer-to-bead ratio was between 6.4 × 104 and 1.28 × 105, where the rate increase was more than twice.

### 3.4. Confirmation of Aptamer-to-Bead Ratio by Analysis of Free Aptamer Concentration

**Figure 4:**
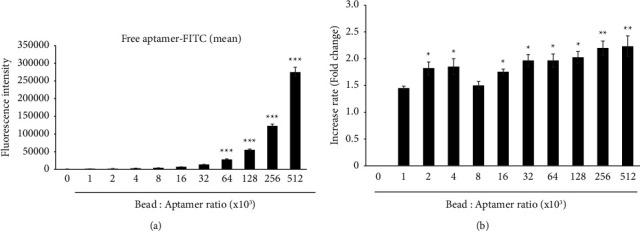
Determination of the most efficient aptamer-to-bead ratio by varying the free aptamer concentration. (a) Analysis of the amount of free aptamer by fluorescence spectrometry. “0” indicates control without aptamer. Error bars represent standard deviations of replicates (*n* = 3). ^*∗∗∗*^ Statistically significant difference in fluorescence intensity compared to control (*P* value <0.001). (b) Bar graph representing the rate of fluorescence increase measured under the same conditions as in Figure 2(c). The statistically significant difference in fluorescence intensity compared to the ratio 1 × 10^3^. ^*∗*^*P* < 0.05,^*∗∗*^*P* < 0.01. Without a star, no statistical significance.

The results from the flow cytometry and fluorescence analysis suggested that it is not effective to continue increasing the aptamer-to-bead ratio beyond a certain value. To confirm this hypothesis, we analyzed the concentration of free aptamer that remained after exposure of the aptamer to the beads for complexation. The concentration of the free aptamer gradually increased depending on the initial amount of aptamer (Figure 4(a)). At low aptamer-to-bead ratios (<6.4 × 104), the increase in the rate of the free aptamer is lower than twice, but at concentrations higher than a ratio of 1.28 × 105, the rate of increase is more than twice (Figure 4(b)). We also found that the rate increase at a ratio of 1.28 × 105 was the highest (Figure 4(b)), indicating that free aptamer molecules are more abundant after excessive aptamer-bead interaction. In addition, our data suggest that an excessive concentration of aptamers may result in aptamer molecules interacting with each other, which decreases the efficiency with which they are attached to the beads.

### 3.5. Verification of Aptamer Activity at Each Aptamer-to-Bead Ratio via Inductively Coupled Plasma Optical Emission Spectroscopy (ICP-OES) Analysis

**Figure 5:**
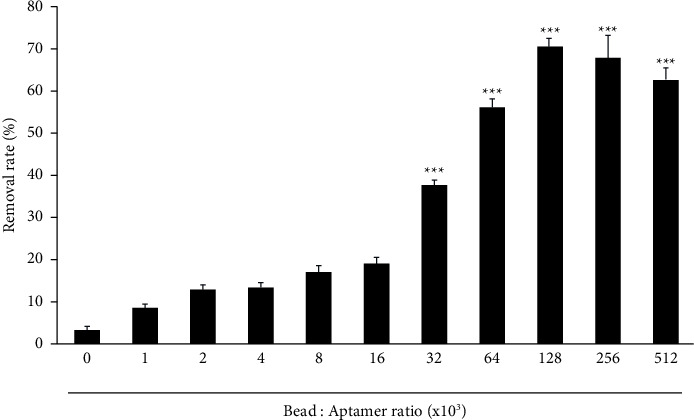
Verification of aptamer Co^2+^ binding capacity at each aptamer-to-bead ratio by ICP/OES analysis. The concentration of Co^2+^ ions was measured by ICP/OES after exposure to the indicated aptamer-bead complex. All experiments were repeated three times. The removal rate is expressed as a percentage (passing through/initial ion concentration). Error bars represent standard deviations of replicates (*n* = 3). ^*∗∗∗*^Statistically significant difference in removal rate compared to control (*P* value <0.001).

The data presented above indicate that binding of the aptamer to the beads was the most effective at a 1.28 × 105 aptamer-to-bead ratio. To verify this result, we determined the aptamer activity at each aptamer-to-bead ratio using ICP-OES analysis. For this test, we used an RNA aptamer that specifically binds Co^2+^ [18] and measured the concentration of Co^2+^ remaining after Co^2+^ binding to the aptamer-bead complex. The rate at which Co^2+^ was removed by the aptamer-bead complex was the highest at the aptamer-to-bead ratio of 1.28 × 105 (Figure 5), showing a pattern similar to our results described above. Although the aptamer-to-bead ratio of 2.56 × 105 was the highest, as determined by flow cytometry and fluorescence analyses (Figures 2 and 3), the aptamer-to-bead ratio was the most effective between 1.26 and 2.56 × 105 in experiments to remove real targets (cobalt), as shown in Figure 5. We also conducted experiments with various aptamers of similar lengths to see if the optimal ratio derived from this experiment could be applied to other aptamers (Supplementary Table). The experiments confirmed that the results of the paper are applicable to similar aptamers with the same size beads (Supplementary Figure). These results indicate that the use of flow cytometry and fluorescence analysis to determine the aptamer-to-bead ratio is sufficiently accurate. Several factors, such as the bead size, aptamer length, and buffer conditions, need to be considered when research involving the use of an aptamer is first started. The method proposed in this study is therefore useful in that it enables the optimal aptamer-to-bead ratio to be determined under various initial conditions.

## 4. Conclusion

In conclusion, we developed a new method that relies on flow cytometry to determine the effective coupling ratio between aptamers and beads. Although it is important to have as many aptamers attached to the beads as possible, the effectiveness of the aptamer-bead complexes is more important. In this respect, this study determined the most effective aptamer-to-bead ratio using flow cytometry and various other methods. Flow cytometry analysis revealed that an aptamer-to-bead ratio of 2.56 × 105 enables the largest amount of aptamer to bind to the beads. We also found that an aptamer-to-bead ratio higher than 2.56 × 105 significantly reduced the amount of aptamer attached to the beads. This finding was corroborated using other fluorescence techniques. In addition, our measurements of the activity of bound aptamers confirmed that an excessively large ratio of aptamer to beads (e.g., 5.12 × 105), has the effect of decreasing the binding efficiency between the aptamer and the target. This experimental method is expected to be useful for determining the combination of various aptamers and beads in the future.

## Data Availability

The data used to support the findings of this study are within the paper.

## Supplementary Materials

Supplementary Materials Supplementary Table. Aptamers sequence and length. The table shows the information for RNA aptamer N1 used in the FACS experiment. And information about the aptamer used to ensure that the aptamer bead ratio derived in the paper is applicable to aptamers of the same or similar length as RNA aptamer N1. Supplementary Figure. Verification of aptamers binding capacity. Verification of N1-2174 (a), FA-N3 (b), and FA-M3 (c) aptamer Ni2+ binding capacity at each aptamer-to-bead ratio by ICP/OES analysis. The concentration of ions was measured by ICP/OES after exposure to the indicated aptamer-bead complex. All experiments were repeated three times. The removal rate is expressed as a percentage (passing through/initial ion concentration).
