# Supplementary material for: Method to Determine the Optimal Aptamer-to-Bead Ratio by Using Flow Cytometry
Source: Scientifica (Cairo). 2023 Jul 11;2023:5842652. doi: 10.1155/2023/5842652 (PMC10353897; doi:10.1155/2023/5842652)
Supplement: Supplementary Materials — Supplementary Table. Aptamers sequence and length. The table shows the information for RNA aptamer N1 used in the FACS experiment. And information about the aptamer used to ensure that the aptamer bead ratio derived in the paper is applicable to aptamers of the same or similar length as RNA aptamer N1. Supplementary Figure. Verification of aptamers binding capacity. Verification of N1-2174 (a), FA-N3 (b), and FA-M3 (c) aptamer Ni2+ binding capacity at each aptamer-to-bead ratio by ICP/OES analysis. The concentration of ions was measured by ICP/OES after exposure to the indicated aptamer-bead complex. All experiments were repeated three times. The removal rate is expressed as a percentage (passing through/initial ion concentration). [file 5842652.f1.zip › Supplementary Table.docx]

**Table S. Aptamers sequence and length.** The table shows the information for RNA aptamer N1 used in the FACS experiment. And information about the aptamer used to ensure that the aptamer bead ratio derived in the paper is applicable to aptamers of the same or similar length as RNA aptamer N1.

| **Aptamer** | **Sequence** | **Length**  **(bp)** | **Binding**  **target** |
| --- | --- | --- | --- |
| RNA N1 | GGGAGAGGAUACUACACGUGAUAGUCAGGGAACAUGACAAACACAGGGACUUGCGAAAAUCAGUGUUUUGCCAUUGCAUGUAGCAGAAGCUUCCG | 95 | Co^2+^ / Ni^2+^ |
| RNA N1 2174 | GCUGCAAAACGUCGGCAGGGAUAGUCAGGGAACAUGACAAACACAGGGACUUGCGAAAAUCAGUGUUUUGCCAUCCCAGCCGAGGGACCGCAGCG | 95 | Co^2+^ / Ni^2+^ |
| DNA FA-N3 | GGTAATACGACTCACTATAGGGAGATACCAGCTTATTCAATTTCCGGTCACGGAGGTAGACTACTGTCGTTGGCGGTGGTCGAGATTGCACTTACTATCT | 100 | Ni^2+^ |
| DNA FA-M3 | GGTAATACGACTCACTATAGGGAGATACCAGCTTATTCAATTAGAGGTCAGGGTAGGGAGGGGGGAATAAGGTGTCACACGGAGATTGCACTTACTATCT | 100 | Mn^2+^ |
